# Supplementary material for: Novel Soil Bacterium Strain Desulfitobacterium sp. PGC-3-9 Detoxifies Trichothecene Mycotoxins in Wheat via De-Epoxidation under Aerobic and Anaerobic Conditions
Source: Toxins (Basel). 2020 Jun 1;12(6):363. doi: 10.3390/toxins12060363 (PMC7354494; doi:10.3390/toxins12060363)
Supplement: Supplementary file 1 [file toxins-12-00363-s001.pdf]

Supplementary Materials: Novel Soil Bacterium Strain Desulfitobacterium sp. PGC-3-9 Detoxifies Trichothecene Mycotoxins in Wheat via De-Epoxidation under Aerobic and Anaerobic Conditions

Wei-Jie He, Meng-Meng Shi, Peng Yang, Tao Huang, Qing-Song Yuan, Shu-Yuan Yi, Ai-Bo Wu, He-Ping Li, Chun-Bao Gao, Jing-Bo Zhang and Yu-Cai Liao

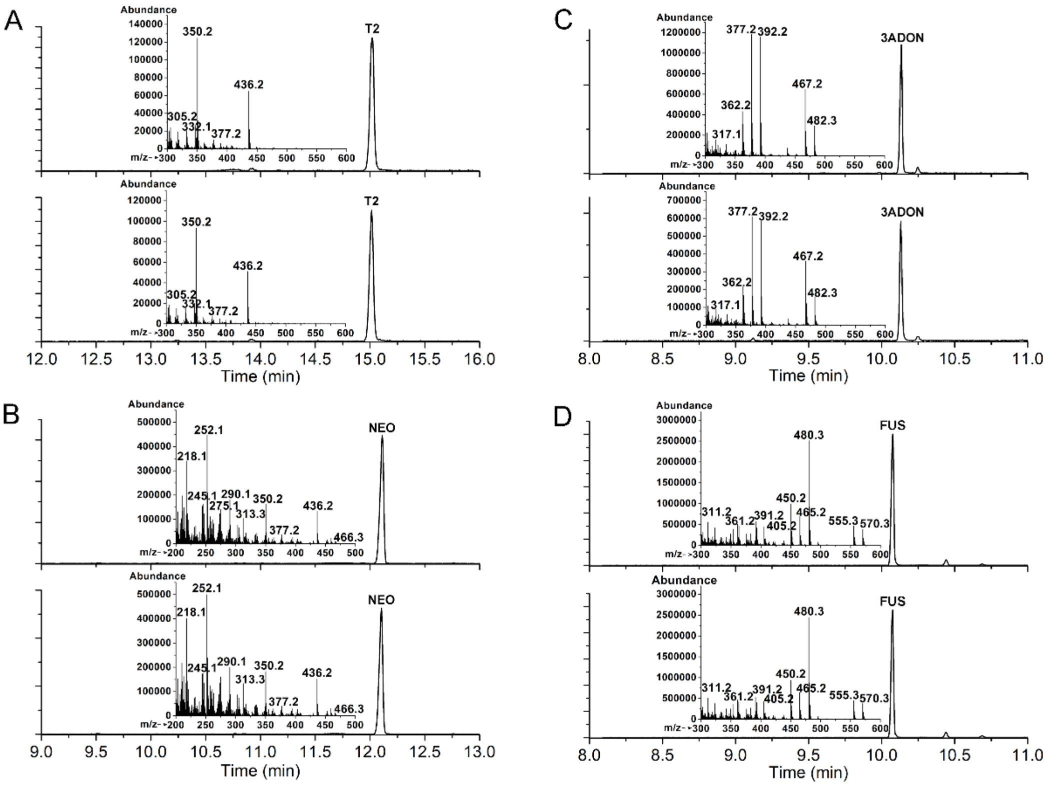

Figure S1. GC-MS chromatography of trichothecene mycotoxins before and after incubation with strain PGC-3-9. (**A**–**D**) GC-MS chromatography of trichothecenes T2 (**A**), neosolaniol (**B**; NEO), 3-Acetyl deoxynivalenol (**C**; 3ADON) and Fusarenon X (**D**; FUS) before and after incubation with PGC-3-9. Total ion chromatograms and mass spectra of each trichothecene mycotoxin before (upper panel) and after (lower panel) incubation with PGC-3-9. Detailed mass spectra of mycotoxins before and after incubation are illustrated as small charts within the upper and lower panels.
